# Leukocyte telomere length and mortality risk in patients with type 2 diabetes

**DOI:** 10.18632/oncotarget.10615

**Published:** 2016-07-15

**Authors:** Anna Rita Bonfigli, Liana Spazzafumo, Francesco Prattichizzo, Massimiliano Bonafè, Emanuela Mensà, Luigina Micolucci, Angelica Giuliani, Paolo Fabbietti, Roberto Testa, Massimo Boemi, Fabrizia Lattanzio, Fabiola Olivieri

**Affiliations:** ^1^ Scientific Direction, INRCA-IRCCS National Institute, Ancona, Italy; ^2^ Center of Biostatistics, INRCA-IRCCS National Institute, Ancona, Italy; ^3^ Institut d'Investigacions Biomèdiques August Pi i Sunyer (IDIBAPS), C/Rosselló, Barcelona, Spain; ^4^ Department of Experimental, Diagnostic and Specialty Medicine, DIMES, University of Bologna, Bologna, Italy; ^5^ Center of Clinical Pathology and Innovative Therapy, National Institute INRCA-IRCCS, Ancona, Italy; ^6^ Department of Clinical and Molecular Sciences, DISCLIMO, Università Politecnica delle Marche, Ancona, Italy; ^7^ Experimental Models in Clinical Pathology, INRCA-IRCCS National Institute, Ancona, Italy; ^8^ Metabolic Diseases and Diabetology Unit, INRCA-IRCCS National Institute, Ancona, Italy

**Keywords:** telomere shortening, type 2 diabetes, mortality, aging, Gerotarget

## Abstract

Leukocyte telomere length (LTL) shortening is found in a number of age-related diseases, including type 2 diabetes (T2DM). In this study its possible association with mortality was analyzed in a sample of 568 T2DM patients (mean age 65.9 ± 9 years), who were followed for a median of 10.2 years (interquartile range 2.2). A number of demographic, laboratory and clinical parameters determined at baseline were evaluated as mortality risk factors. LTL was measured by quantitative real-time PCR and reported as T/S (telomere-to-single copy gene ratio). Age, gender, creatinine, diabetes duration at baseline, and LTL were significantly different between T2DM patients who were dead and alive at follow-up. In the Cox regression analysis adjusted for the confounding variables, shorter LTL, older age, and longer disease duration significantly increased the risk of all-cause mortality (HR = 3.45, 95%CI 1.02-12.5, *p* = 0.004). Kaplan-Maier analysis also found a different cumulative mortality risk for patients having an LTL shorter than the median (T/S ≤0.04) and disease duration longer than the median (>10 years) (log-rank = 11.02, *p* = 0.011). Time-dependent mortality risk stratification showed that T2DM duration and LTL combined was a fairly good predictor of mortality over the first 76 months of follow-up.

In conclusion, LTL combined with clinical parameters can provide additive prognostic information on mortality risk in T2DM patients.

## INTRODUCTION

Telomeres are complexes consisting of G-rich DNA sequences and specialized proteins that cap and protect the ends of chromosomes [1]. Telomeric DNA is subject to attrition during mitosis, and replication can go on until a critical threshold of telomere length is reached. Telomeric DNA is also highly prone to oxidative damage, and increases in oxidative stress induce its shortening [2]. For these reasons, telomere shortening is a widely used indicator of replicative senescence and cumulative genomic damage in somatic cells [3, 4], and telomere length, especially leukocyte telomere length (LTL), has extensively been investigated as a biomarker of organismal aging [5–8]. Epidemiological studies have shown that short telomeres are associated with a number of age-related disorders such as cardiovascular and neurodegenerative diseases, cancer [9–13], and metabolic syndrome [14–18]. LTL determination in patients with diabetes mellitus (DM) has highlighted a significant association with the presence and number of diabetes complications [19–21]. Moreover, LTL attrition has also been described in patients with mitochondrial diabetes, suggesting its relevance in the disease [22]. Short leukocyte telomere length predicts risk of diabetes in American Indians [23]. However, a recent study of the US general population has found that LTL is not associated with diabetes [24].

Conflicting data have also been reported on the value of LTL as a predictor of mortality in the general aged population [25–27]. The Leiden Longevity Study and Danish prospective cohort studies have found that LTL is a marker of mortality [28–29], whereas the prospective population-based MrOS-Sweden study has found no such association [27]. Negative results have also been reported in male participants in the Zutphen Elderly Study [30]. As regards the role of LTL as a predictor of mortality in DM patients, a significant association has been described in patients with type 1 Diabetes Mellitus [31], whereas to the best of our knowledge none have been involved patients suffering from type 2 DM (T2DM).

Since in the first quarter of this century the prevalence of DM is likely to double, managing such patients is a crucial clinical and socio-economic issue [32] that makes it all the more urgent to identify new non-invasive prognostic biomarkers and mortality-associated risk factors for T2DM. Yet, despite the key importance of the issue, none of the innovative biomarkers proposed to date appear to be clinically effective [33–34].

This study was devised to explore whether LTL, combined with a number of demographic, laboratory, and clinical parameters, has predictive value for all-cause mortality in a large group of T2DM patients.

## RESULTS

The baseline characteristics of participants are listed in relation to survival status at the end of follow-up (Table 1). Age, gender, creatinine value, presence of complications, diabetes duration at baseline (expressed as years from diagnosis) and LTL (measured as T/S) were significantly different between patients who were alive or dead at the end of the study.

**Table 1:** Characteristics of T2DM patients divided by survival status at the end of follow-up

	Alive (*n* = 453)	Dead (*n* = 88)	p*
**Age**	65.1 ± 7.9	70.4 ± 7.2	**<0.001**
**Males**	203 (52.0)	57 (64.0)	**0.036**
BMI, kg/m^2^	28.6 ± 4.4	28.9 ± 5.5	0.157
Smokers no. (%)	36 (13.8)	13 (14.8)	0.773
Hypertension, no. (%)	252 (56.0)	60 (65.5)	0.057
**Presence of complications**	194 (49.6)	61 (68.5)	**0.001**
Fasting glucose, (mg/dl)	162.7 ± 48.1	170.0 ± 51.8	0.083
HbA1C, %	7.5 ± 1.2	7.7 ± 1.3	0.059
White blood cells, 10^3^/l	6.6 ± 1.5	6.9 ± 1.8	0.087
Red blood cells	4.7 ± 0.4	4.6 ± 0.5	0.149
Hs-CRP(mg/dl)	4.0 ± 5.3	6.6 ± 10.5	0.073
**Creatinine (mg/dl)**	0.9 ± 0.3	1.0 ± 0.5	**<0.001**
Uric acid (mg/dl)	4.7 ± 1.2	4.9 ± 1.2	0.172
Total cholesterol (mg/dl)	205 ± 38	203 ± 41	0.681
HDL cholesterol (mg/dl)	52.3 ± 13.7	49.6 ± 15.9	0.116
Triglycerides (mg/dl)	133.8 ± 84.6	160.8 ± 136.6	0.095
**LTL (T/S)**	**0.45 ± 0.21**	**0.39 ± 0.15**	**0.004**
**Diabetes duration, years**	**14.0 ± 10.9**	**19.7 ± 12.0**	**<0.001**

Variables are expressed as mean (SD) for normally distributed variables and as number (percentage) for categorical variables. BMI: body mass index; HbA1C glycated hemoglobin: Hs-CRP; high-sensitivity C-reactive protein; LTL: Leukocyte telomere length; T/S: telomere-to-single copy gene ratio.

Since significantly different creatinine values were found in patients with and without diabetes complications (*t*-test = −5.042; *p* < 0.001), only the presence of complications was considered in subsequent analyses.

LTL was inversely related to age *r* = −0.15; *p* < 0.01) and was greater in women than in men, although the difference was not significant as a consequence all further analyses were adjusted for age and gender.

**Figure 1:**
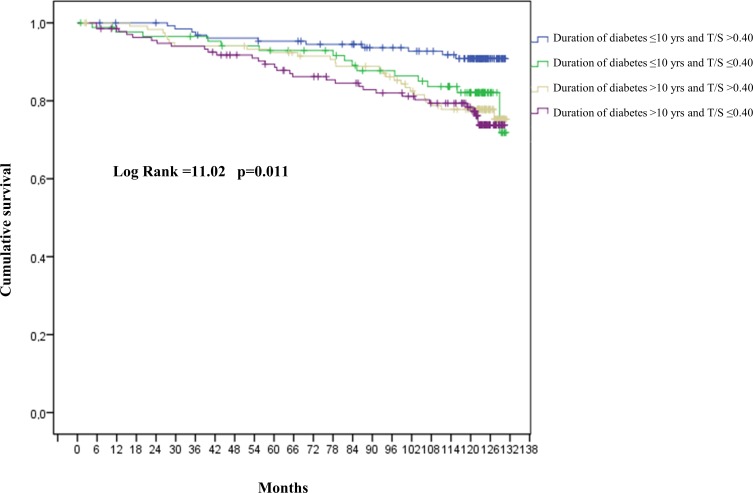
Cumulative risk of mortality for T2DM patients T/S: telomere-to-single copy gene ratio. Patients with diabetes duration ≤ 10 years and T/S > 0.40 were considered as the reference group.

**Figure 2:**
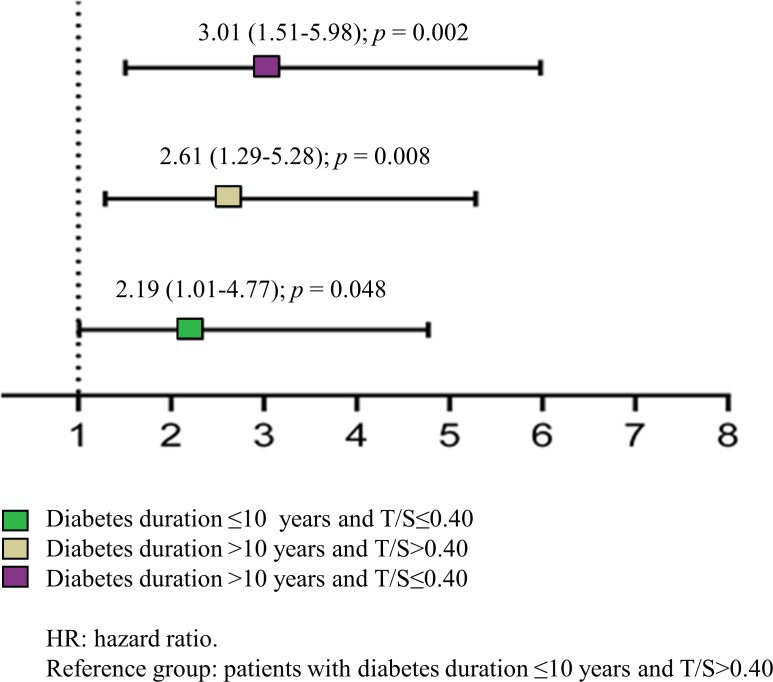
Mortality risk of T2DM patients grouped by disease duration and LTL LTL: Leukocyte telomere length; T/S: telomere-to-single copy gene ratio.

The predictive value of LTL - tested by Cox regression analysis including age, gender, presence of diabetes complications, T2DM duration and LTL as continuous variables (Table 2) - disclosed that a shorter LTL predicted all-cause mortality in the three models. In the unadjusted model the hazard ratio (HR) was 5.65 and the 95% confidence interval (CI) was 1.61-19.81 (*p* = 0.007); in the analysis adjusted only for diabetes complications data were as follows: HR = 4.73; 95% CI 1.29-17.39, *p* = 0.004, and in the model adjusted for diabetes complications, age, gender and disease duration data were: HR = 3.45; 95% CI: 1.02-12.5, *p* = 0.004. Since only age and T2DM duration were significantly associated with an increased risk of mortality in the third model, Kaplan-Maier analysis was performed on T2DM patients in relation to disease duration and LTL. Patients were divided by median disease duration (10 years) and LTL (T/S = 0.40). Comparison of survival distribution in the 4 groups yielded a significant difference (log-rank = 11.2; *p* = 0.011) (Figure 1). Patients with diabetes duration ≤ 10 years and T/S > 0.40 were selected as the reference group; the other three groups were characterized by T2DM duration ≤ 10 years and T/S ≤ 0.40 (group A); T2DM duration > 10 years and T/S > 0.40 (group B); and T2DM duration > 10 years and T/S ≤ 0.40 (group C). Comparison with the reference group demonstrated a progressive increase in the cumulative risks of mortality in the three groups: group A, HR = 2.19, 95% CI 1.01-4.77, *p* = 0.048; group B, HR = 2.61 95% CI 1.29-5.28, *p* = 0.008; and group C, HR = 3.01 95% CI 1.51-5.98, *p* = 0.002 (Figure 2).

**Table 2:** Hazard ratios related to telomere length in T2DM patients

	Unadjusted HR (95% CI)	Adjusted HR (95% CI)	
LTL (T/S)	5.65 (1.61-19.81)	4.73 (1.29-17.39)	3.45 (1.02-12.5)
Presence of complications		1.25 (0.78-2.00)	0.94 (0.58-1.52)
Age			1.08 (1.04-1.12)
Males			1.46 (0.93-2.3)
Diabetes duration			1.52 (1.01-2.43)

T/S: telomere-to-single copy gene ratio. HR: hazard ratio. CI: confidence interval.

Bold: significant variables, *p* < 0.05.

**Figure 3:**
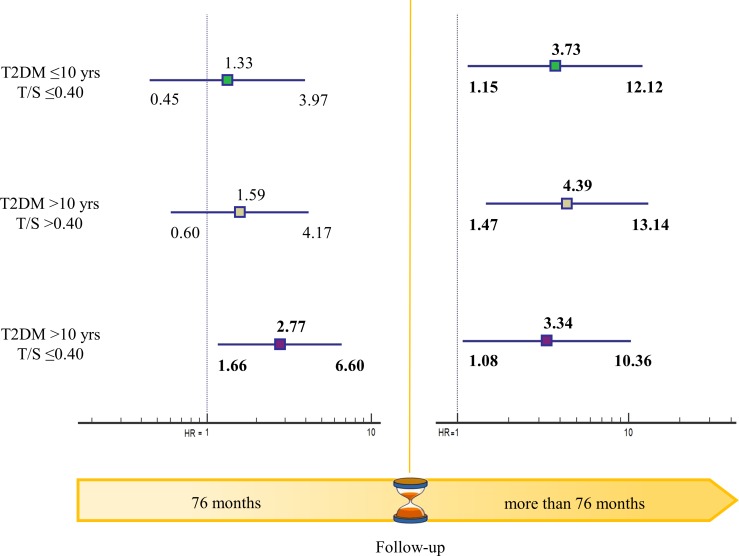
Time-varying mortality risk in T2DM patients grouped by disease duration and LTL LTL: Leukocyte telomere length; T/S: telomere-to-single copy gene ratio.

The time-varying risk of mortality was obtained by calculating the month when half of the patients had died (76^th^ month) and splitting the follow-up into the time up to 76 months and the remaining period. In the first 76 months, the mortality risk of patients with T2DM duration > 10 years and T/S ≤ 0.40 was increased compared with the reference group (HR = 2.77, 95% CI: 1.66-6.60, *p* < 0.05). In the remaining months the mortality risk compared with the reference group was increased in all the other groups (Figure 3).

**Figure 4:**
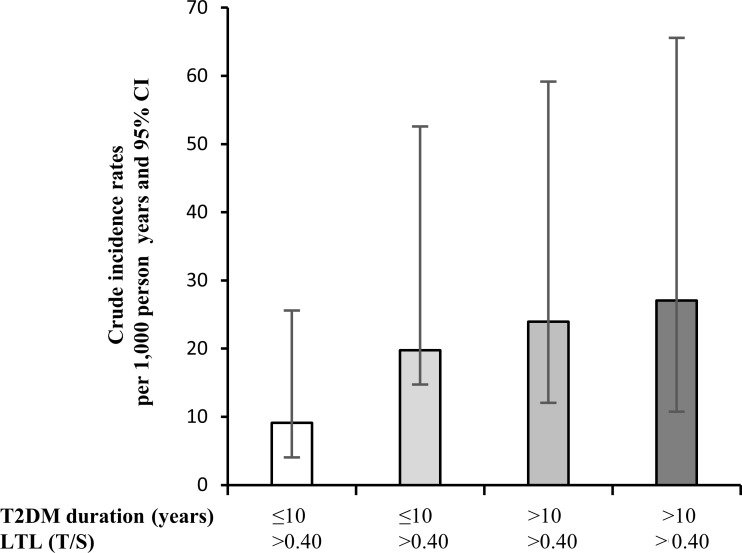
Crude incidence mortality rates per 1,000 person years in T2DM patients grouped by LTL and disease duration LTL: Leukocyte telomere length; T/S: telomere-to-single copy gene ratio.

The crude mortality rate rose from the reference group to the group with T2DM duration > 10 years and T/S ≤ 0.40 (9 *vs.* 27 dead per 1,000 person years respectively) (Figure 4).

## DISCUSSION

The present data, found in a large sample of Caucasian patients with T2DM followed for a median of 10.2 years, highlighted a complex relationship among LTL, diabetes duration, and mortality. Since T2DM is a progressive disease, patients with longer standing disease clearly showed higher mortality rates than those with shorter diabetes duration [35]. The novel finding was that T2DM duration and LTL combined was a fairly good predictor of mortality, since patients with shorter LTL and longer disease duration at baseline had an increased mortality risk during follow-up than those with longer LTL and shorter T2DM duration.

Chronological age is indisputably the major risk factor for death. However, aging research has experienced an unprecedented advance over recent years with the discovery that to improve human health during aging is necessary to slow the “rate of aging”. Since the rate of aging differs between subjects, chronological age cannot accurately define the functional abilities of tissues and organs during life, expecially in the latest period of life, when healthy/unhealthy phenotypes clearly identify people with different rate of aging [36]. Therefore, there is an urgent need to identify new parameters able to measure the “rate of aging”, to better predict health-span and illness/death [37]. Among biomarkers strongly association with risk of illness and death, epigenetic markers are those that better reflect the interaction between personalized genetic background and environmental factors and are therefore useful to define the ability of body to cope with stressors at any times [38]. In this scenario LTL become an “epigenetic biomarker” that could contribute to define “the rate of aging” and therefore contribute to improve mortality risk prediction. LTL shortening is a well-established marker of cellular senescence and can directly promote accelerated leukocyte aging. Although investigations into T2DM pathophysiology commonly focus on insulin resistance, and impaired insulin secretion, recent data suggest a broader range of causes. Since T2DM has begun to be considered as a model of “accelerated aging” [39, 40], LTL shortening has been hypothesized to be indicative of senescent cell accumulation and of an increased burden of systemic inflammation [41]. Although inflammation has been suggested to have a key role in T2DM development and progression, its molecular bases are still being investigated [42–44]. Inflammaging - the age-related chronic state of low grade inflammation that modulates the aging process and promotes the development and progression of age-related diseases - is mainly sustained by protracted activation of the immune system and an increased burden of senescent cells with a secretory phenotype (SASP) [45–46]. Support for the link between telomere length and systemic inflammation has recently been provided by the report that telomeric repeat-containing RNA (TERRA) is a component of extracellular inflammatory exosomes [47]. Since telomere shortening induces TERRA expression and TERRA-enriched exosome stimulate the release of inflammatory cytokines from human peripheral blood mononuclear cells and fibroblasts, telomere shortening have the potential to promote a proinflammatory environment [47–48].

However, the *in vivo* relevance of the buildup of senescent cells in diabetes has not yet been demonstrated. Therefore, the question whether glucose-related metabolic changes promote telomere attrition, or whether accelerated cellular senescence plays a large pathogenic role in T2DM development and progression, is still outstanding [43]. Senescent cells might be part of a pathogenic loop in diabetes as both the cause and consequence of metabolic alterations. We recently hypothesized that an increased number of senescent cells can contribute to spread senescence at the systemic level, promoting diabetes progression [46]. The significantly shorter LTL measured in T2DM patients compared with healthy subjects over a wide age range has lent support to the hypothesis, which is also consistent with the beta cell failure and the increased senescence of vascular endothelial and smooth muscle cells seen in hyperglycemic media [49]. The notion is further reinforced by the inverse age-dependent relationship documented between LTL and insulin resistance [50]. Notably, *in vivo* evidence of increased senescence in both atherosclerotic plaques and renal tissue of diabetes patients suggest the relevance of this phenomenon to the vascular complications of diabetes [20, 50]. Increased beta cell senescence in diabetic patients has also been demonstrated [51]. Telomere length shortening was recently observed not only in leukocytes of T2DM patients but also in pancreatic β-cells, suggesting an impaired capacity of proliferation and insulin secretion [52]. Hyperglycemia, oxidative stress, and telomere attrition in pancreatic β-cells and adipocytes seem to create a vicious cycle that underlies the pathophysiology of T2DM.

Since novel pharmacological approaches targeting senescent cells have been proposed for the prevention of T2DM and its complications, it is of the utmost importance to establish whether disease duration and the development of diabetes complications correlate with the burden of senescent cells as measured by LTL. Moreover, a rapidly growing body of research has been showing that telomere length and attrition rate can be directly affected by environmental and lifestyle factors, suggesting that LTL could be useful to monitor the effectiveness of lifestyle-based interventions or drug treatments directed at modifying the mortality risk of diabetic patients.

In conclusion, the present findings support the notion that LTL could be a surrogate biomarker to quantify systemic senescence status and inflammaging.

### Limitations of the study

This is a predictive study. To estimate the prognostic relevance of LTL on T2DM patient mortality, the present data need to be validated in an independent T2DM sample.

## MATERIALS AND METHODS

### Patients

A total number of 568 patients with T2DM were recruited at the Italian National Research Center on Aging (INRCA), Ancona (central Italy), from 2003 to 2006. Their mean age was 65.7 ± 8.2 years. All patients gave their informed written consent to participate in the study. The study protocol and the informed consent procedure were approved by the INRCA Institutional Ethics Committee and were in line with the Declaration of Helsinki.

Inclusion criteria were a diagnosis of T2DM according to ADA criteria [53], a body mass index (BMI) < 40 kg /m^2^; age 35-85 years, T2DM diagnosis at least a year previously, and the ability and willingness to provide the written informed consent. The information collected included baseline vital signs, anthropometric factors, medical history and behaviors, and physical activity.

The presence/absence of diabetes complications was established as follows: diabetic retinopathy, by fundoscopy through dilated pupils and/or fluorescence angiography; renal impairment, as an estimated glomerular filtration rate < 60 ml/min per 1.73 m^2^; neuropathy, by electromyography; ischemic heart disease by clinical history and/or ischemic electrocardiographic alterations; and peripheral vascular disease, including atherosclerosis obliterations and cerebrovascular disease, based on history, physical examination and Doppler velocimetry. No complication were documented in 274 patients, whereas at least one complication was found in 294 participants; in particular, 103 patients suffered had neuropathy, 74 had nephropathy, 21 had kidney failure, 156 had retinopathy, 35 had chronic lower limb arterial disease, and 100 had carotid arterial disease, cerebrovascular and ischemic heart disease (MACE). Several patients suffered from multiple complications.

Participants were classified as hypertensive if they had been prescribed antihypertensive medication or had a systolic blood pressure > 140 mmHg and/or a diastolic blood pressure > 90 mmHg, measured in seated position on at least three different occasions. Overnight fasting venous blood samples drawn from 08.00 to 09.00 h were obtained from all participants and analyzed immediately or stored at −80°C until they were assayed. Median follow up time was 10.2 years (interquartile range 2.2), when patients were recorded as being alive or dead. The last information on vital status was obtained in May 2014. Overall survival was defined as the time between enrolment and death; for patients who were alive at the end of follow-up, survival time was set at the time of the last available assessment. The survival status of 27 patients were not found and the dropout represents the 4.7% of the sample.

The mean baseline age of patients without complications who were alive or dead at the end of follow-up was respectively 64.0 ± 8.0 and 66.4 ± 7.4 years; the mean baseline age of those with complications was respectively 69.0 ± 8.7 and 70.0 ± 6.7 years. Among patients without complications who were alive or dead at the end of follow-up, men were respectively 76 (41.3%) and 19 (65.5%), and among those with complications they were respectively 129 (61.4%) and 34 (61.8%).

By the end of the study 89 patients, 28 (13%) without and 61 (20.7%) with complications (*p* < 0.01), had died; among the latter, 22 had suffered from neuropathy, 18 from nephropathy, 9 from kidney failure, 36 from retinopathy, 11 from chronic lower limb arterial disease, and 22 from MACE.

At the time of enrolment, 48.1% of patients were receiving sulfonylurea, 36.4% biguanides, 17.8% insulin combined with an oral anti-diabetes medication, and 2.3% thiazolidinediones.

### Laboratory assays

Fasting blood concentrations of glucose, glycated hemoglobin (HbA1c), white and red blood cells, high-sensitivity C-reactive protein (hs-CRP), creatinine, urea nitrogen, uric acid, total and HDL cholesterol, and triglycerides were measured by standard procedures according to each manufacturer's specifications. The value of all parameters was in the recommended range.

### Measurement of telomere length

High molecular weight DNA was isolated from white blood cells using a Qiagen DNA extraction kit (Milano, Italy). Telomere length was measured and compared as telomere-to-single copy gene ratio (T/S) using quantitative real-time PCR as described by Cawthon et al. [54], with some modifications using a 5 μl aliquot containing 20 ng DNA and 10 μl of master mix per patient. For each standard curve, one reference DNA sample was diluted serially in water by 1.68-fold dilution to produce 5 DNA concentrations ranging from 30 to 2 ng in 5 μl. To reduce interassay variability, the telomere and the single-copy gene (36B4) were analyzed on the same plate. Primer sequences and concentrations for telomere and 36B4 were as described by Cawthon et al. [54].

The thermal cycling profile was: (1) one cycle of 10 s at 95 ^°^C; (2) 30 cycles of 5 s at 95 ^°^C, 15 s at 57 ^°^C and 20 s at 72 ^°^C. Measurements were performed in duplicate and reported as T/S ratio relative to a calibrator sample (Roche, Milano, Italy) to allow comparison across runs. The real-time Chromo4 MJ Research system (Bio-Rad Laboratories, Hercules, CA, USA) was used for all PCRs.

The coefficients of variation within duplicates of the telomere and single-gene assay were 2% and 1.8%, respectively. Approximately 30% of samples were repeated on different plates to assess T/S measurement reproducibility. The interassay coefficient of variation was < 10%. All analyses were blind. The correlation coefficient between T/S and the telomere restriction fragment was R^2^ = 0.88.

### Statistical analysis

Biochemical and clinical data are reported as means (± SD) for normally distributed variables and as number and percentage for categorical variables. The characteristics of patients, divided by survival status (alive/dead) at the end of follow-up, were compared using the t-test for continuous variables and the chi-square test for categorized variables. The predictive value of LTL was tested using unadjusted Cox regression analysis with telomere length as a continuous variable. Thereafter, the model was adjusted for the presence/absence of complications and, in an additional model, for the presence/absence of complications, age, gender and diabetes duration.

Kaplan-Meier analysis was applied to assess the combined effect of diabetes duration and LTL by dividing patients into 4 prognostic groups in relation to median disease duration (10 years) and LTL (T/S = 0.40). Patients with ≤ 10 years disease duration and T/S > 0.40 were the reference group. Calculation of the month when half of the patients had died (76^th^ month) allowed evaluating the time-varying risk of mortality by splitting the follow-up into the time up to 76 months and the remaining period.

The crude incidence mortality rate was calculated and reported as number of patients who had died per 1,000 patient years.

A probability value < 0.05 was considered statistically significant.

Data were analyzed with SPSS/Win program (version 21.0; Spss Inc., Chicago, IL, USA).
